# Comparison of efficacy and safety of conventional laparoscopic radical prostatectomy by the transperitoneal versus extraperitoneal procedure

**DOI:** 10.1038/srep14442

**Published:** 2015-10-13

**Authors:** Cao De Hong, Liu Liang Ren, Wei Qiang, Wang Jia, Hu Ying Chun, Yang Lu, Liu Zheng Hua, Li Heng Ping, Yan Shi Bing, Li Yun Xiang

**Affiliations:** 1 From the Department of Urology, West China Hospital of Sichuan University, Chengdu, China; 2 From the Department of Geriatrics, West China Hospital of Sichuan University, Chengdu, China; 3 From the Department of Urology, Gansu Provincial Hospital, Lanzhou, China

## Abstract

Worldwide, prostate cancer (PCa) is the second most common malignancy in males. We undertook a meta-analysis to compare the efficacy and safety of conventional laparoscopic radical prostatectomy with a transperitoneal (TLRP) approach, versus that of an extraperitoneal (ELRP) approach, for treatment of localized PCa. A comprehensive literature search retrieved 14 publications, with a total of 1715 patients. Meta-analysis of these studies showed that an ELRP approach was associated with a significantly shorter postoperative catheterization time (MD: 1.99; 95% CI: 0.52 to 3.54; P = 0.008), less blood transfusion rate (OR: 2.05; 95% CI: 1.03 to 4.06; P = 0.04), shorter intestinal function recovery time (MD: 0.08; 95% CI: 0.52 to 1.09; P < 0.0001) and shorter hospitalization days (MD: 2.71; 95% CI: 1.03 to 4.39; P = 0.002). In addition, our results showed no statistically significant differences between the two groups in operation time (MD: 19.39; 95% CI: −6.67 to 45.44; P = 0.014), intraoperative blood loss (MD: 4.89; 95% CI: −105.00 to 114.79; P = 0.93) and total complication rate (RR: 1.22; 95% CI: 0.86 to 1.74; P = 0.27). In summary, our meta-analysis showed that ELRP is likely to be a safe and feasible alternative for localized PCa patients compared with TLRP.

Prostate cancer (PCa) is the second most common malignancy in males worldwide, and has increased in incidence in recent years^1^. In 2014, PCa incidence rates in the United States reached approximately 27% of all new cancer cases^2^. Radical prostatectomy is one of the methods in treating localized PCa^3^. A minimally invasive technique, conventional laparoscopic radical prostatectomy (LRP) was first introduction in 1992 by Schuessler *et al.*^4^; LRP has since become a common treatment approach for the management of patients with localized PCa^5^.

However, both approaches to conventional LRP are routinely used in urological practice: the transperitoneal LRP (TLRP) and the extraperitoneal LRP (ELRP). Both approaches have been touted as efficacious and safe techniques by which to perform LRP^6^,^7^,^8^,^9^,^10^,^11^; however, whether TLRP is superior to ELRP in terms of efficacy and safety has become a subject of controversy in recent years. We performed a meta-analysis to assess the safety and efficacy of TLRP and ELRP.

## Results

**Figure 1:**
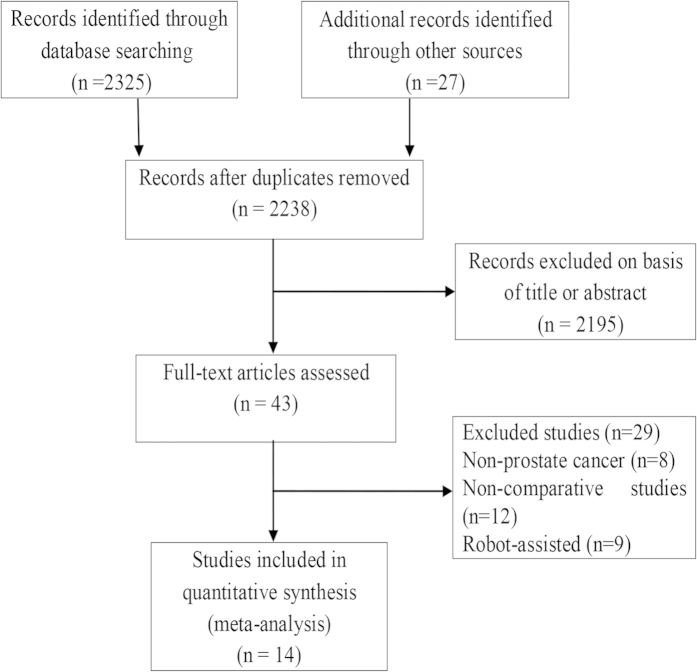
Flow diagram of evidence acquisition.

We formulated a comprehensive search strategy to identify all relevant studies, regardless of the language or publication status. Through literature selection, 14 studies were identified from the literature search. The literature screening process is summarized in Fig. 1.

A total of 1715 patients were included in the 14 studies^7^,^8^,^9^,^10^,^11^,^12^,^13^,^14^,^15^,^16^,^17^,^18^,^19^,^20^; of these, 939 underwent TLRP and 776 underwent ELRP. All included studies reported on the number of patients. Two studies failed to report mean PSA levels^12^,^14^. Seven studies reported on the mean Gleason scores^7^,^9^,^10^,^11^,^16^,^18^,^19^. The basic characteristics and quality assessments of the included studies were summarized in Table 1.

### Study results and meta-analysis

#### Operation time (minutes)

**Figure 2:**
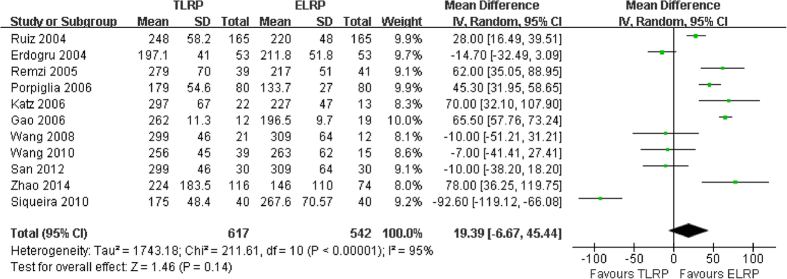
Forest plot of operation time between TLRP and ELRP group.

Operation time was reported in eleven studies^9^,^10^,^11^,^13^,^14^,^15^,^16^,^17^,^18^,^19^,^20^ (1159 patients): 617 receiving TLRP and 542 receiving ELRP. Heterogeneity was observed in the pooled analysis (P < 0.0001; I^2 ^= 95%). In this present meta-analysis of the eleven studies using the random-effect model, the pooled estimates showed no statistically significant difference between the two groups (mean difference [MD]: 19.39; 95% confidence interval [95% CI]: −6.67–45.44; P = 0.014) (Fig. 2). However, after excluding the results from Siqueira *et al.*^17^, sensitivity analysis showed that there was a statistically significant difference between the TLRP and ELRP groups (MD: 30.75; 95% CI: 9.66–51.84; P = 0.004).

#### Intraoperative blood loss (mL)

**Figure 3:**
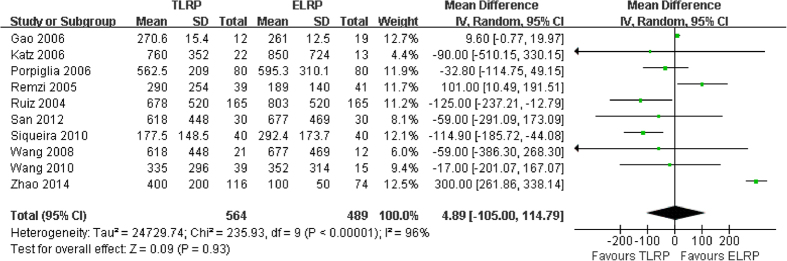
Forest plot of intraoperative blood loss between TLRP and ELRP group.

Mean blood loss during LRP surgery was reported in ten studies^9^,^11^,^13^,^14^,^15^,^16^,^17^,^18^,^19^,^20^ (1053 participants). Heterogeneity was detected in the pooled analysis (P < 0.0001; I^2^ = 96%). Data from the ten studies were merged for meta-analysis using a random-effects model. The result revealed no statistically significant difference between the two groups (MD: 4.89; 95% CI: −105.00–114.79; P = 0.93) (Fig. 3). This finding indicates that there was a similar rate of intraoperative bleeding in the TLRP group, compared to the ELRP group.

#### Blood transfusion rate

**Figure 4:**
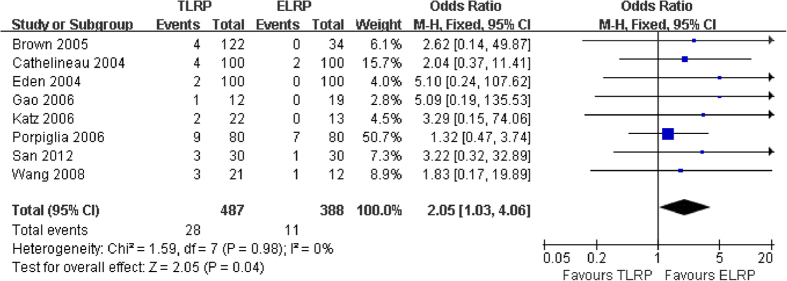
Forest plot of blood transfusion rate between TLRP and ELRP group.

Blood transfusion rate was measured in eight studies^7^,^8^,^12^,^13^,^14^,^15^,^16^,^19^ including 875 patients; our meta-analysis indicated that ELRP group had lower blood transfusion rate than TLRP groups (odd ratio [OR]: 2.05; 95% CI: 1.03 – 4.06; P = 0.04) without statistical heterogeneity (P = 0.98; I^2^ = 0%) (Fig. 4).

#### Postoperative catheterization time (days)

**Figure 5:**
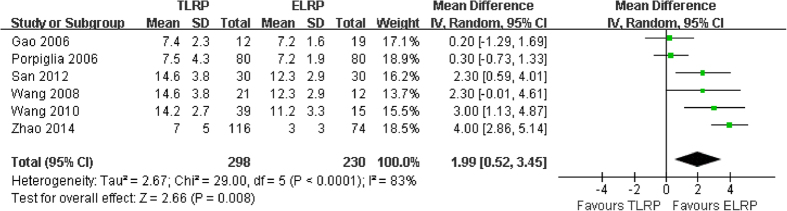
Forest plot of postoperative catheterization time between TLRP and ELRP group.

We extracted the data of postoperative catheterization time from the six studies^13^,^15^,^16^,^18^,^19^,^20^. The pooled data indicated that the heterogeneity was exist (P < 0.0001, I^2 ^= 83%). There was statistically significant in shorter postoperative catheterization time in the ELRP group compared with the TLRP group (MD: 1.99; 95% CI: 0.52–3.54; P = 0.008; Fig. 5).

#### Postoperative intestinal function recovery time (days)

**Figure 6:**
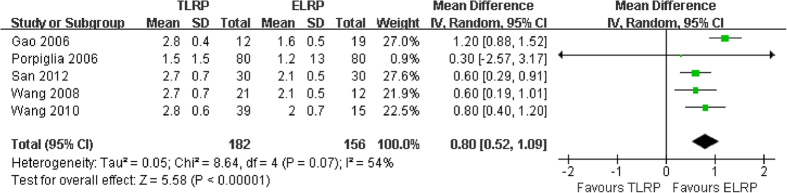
Forest plot of postoperative intestinal function recovery time between TLRP and ELRP group.

Data on postoperative intestinal function time were extracted for meta-analysis from five studies^13^,^15^,^16^,^18^,^19^. Heterogeneity was revealed in the pooled analysis (*P *= 0.07; I^2 ^= 54%); the result from the combined studies using a random-effect model showed that ELRP group was associated with a shorter intestinal function recovery time than TLRP group (MD: 0.80; 95% CI: 0.52–1.09; *P* < 0.0001) (Fig. 6).

#### Hospitalization days

**Figure 7:**
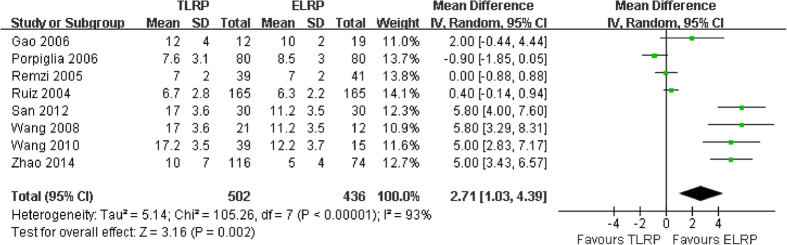
Forest plot of hospitalization days between TLRP and ELRP group.

Eight studies^9^,^11^,^13^,^15^,^16^,^18^,^19^,^20^ including 938 patients compared the hospital time between the TLRP group and ELRP group. High heterogeneity was observed in the pooled analysis (P < 0.0001; I^2 ^= 93%). In the random-effect model meta-analysis of the eight studies, the pooled estimates had statistically significantly different between the 2 groups (MD: 2.71; 95% CI: 1.03–4.39; P = 0.002) (Fig. 7). This pooled analysis indicated that ELRP group was associated with a markedly shorter hospital time than TLRP group.

#### Total complication rate

**Figure 8:**
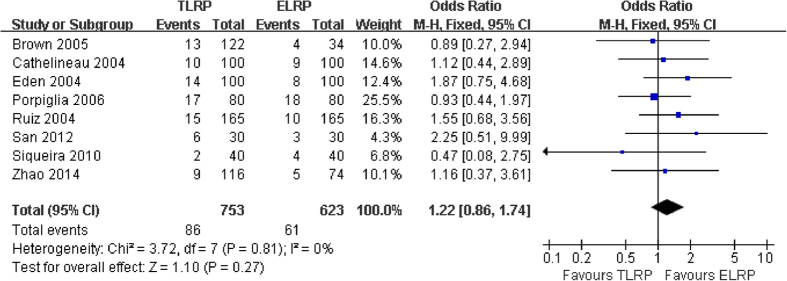
Forest plot of total complication rate between TLRP and ELRP group.

Postoperative total complication rate of the eight studies^7^,^8^,^9^,^12^,^13^,^17^,^19^,^20^ was revealed an 11.4% (86/753) incidence in TLRP group and a 9.8% (61/623) incidence in ELRP group. No heterogeneity was exist in the pooled analysis (*P *= 0.81; I^2 ^= 0%), a fixed-effect model was used for statistical analysis. ELRP group was associated with a lower total complication rate than TLRP group, but the meta-analysis demonstrated that no statistically significant difference was exist (RR: 1.22; 95% CI: 0.86 to 1.74; P = 0.27) (Fig. 8).

## Discussion

Radical prostatectomy is one of the ways for treat patients with localized PCa^3^. The advantages of LRP over open radical prostatectomy include a reduced intraoperative blood loss, a lower blood transfusion rate, shorter hospitalization days, a shorter time to resumption of routine activity and improved scar heals well^21^,^22^. However, robot-assisted radical prostatectomy has higher costs than conventional LRP technology^23^. In current practice, conventional LRP approach for localized PCa patients is available^14^,^15^,^16^,^17^,^18^,^19^,^20^,^21^. In addition, either a transperitoneal or an extraperitoneal approach has been demonstrated to be both safe and effective. Although there is a meta-analysis reporting on the safety and efficacy between TLRP and ELRP that was published in Chinese^24^, this is necessary to update this meta-analysis for provided update evidence regarding their benefits for patients with localized PCa. Therefore, we conduct the systematic review and meta-analysis to compare the safety and efficacy of TLRP with ELRP for the treatment of localized PCa.

The results of the present meta-analysis demonstrated a similar operation time with TLRP, relative to ELRP. Studies conducted by Erdogru *et al.*^10^ and Wang *et al.*^16^ reported that TLRP was associated with a shorter operation time, compared with ELRP. Conversely, other research showed ELRP had a shorter operation time, compared with TLRP^9^,^13^,^14^,^15^. To our surprise, after excluding study data from Siqueira *et al.*^17^ from our meta-analysis, a statistically significant difference was found between the TLRP and ELRP groups. Furthermore, our finding from the pooled analysis revealed that operating times were 30.75 minutes shorter in the ELRP group, compared with the TLRP group; and this difference was statistically significant. The findings from the pooled analysis were consistent with those of 2 studies that were not included in our meta-analysis^21^,^22^. It is plausible that the shorter operative time might result from the direct access to the retropubic space and avoidance of bowel handling in ELRP. We supposed that it is possible that this result was influenced by the small sample sizes or a lack of surgical experience in the ELRP procedure in the study. Therefore, it is necessary to evaluate the operation time of TLRP versus ELRP in further, high quality, randomized controlled trials with larger sample sizes.

Our findings reveal that the average blood loss during TLRP group was similar to that associated with ELRP group, and the observed difference did not achieve statistical significance. Therefore, ELRP had no advantages, in terms of reduced blood loss, compared with TLRP. However, our results regarding blood transfusion rate clearly indicated that the TLRP group had a rate 2.05 times higher than the ELRP group. This present findings also agree with the results of previous controlled clinical trial^15^,^16^.

The present results indicate a significantly faster intestinal function recovery time following ELRP, compared with TLRP. From Fig. 4, it is clear that the mean intestinal function recovery time in the ELRP group was 0.8 times faster than seen in TLRP group. Porpiglia *et al.* reviewed 160 patients who underwent radical prostatectomy, either TLRP or ELRP; they also reported a faster intestinal function recovery time in the ELRP group^13^. Our findings are also consistent with the findings of a previous study by Liu *et al*.^24^.

Additionally, the present meta-analysis demonstrated that ELRP was superior to TLRP with regard to postoperative catheterization time and hospitalization days. However, there was no statistically significant difference in terms of overall complication rate. As described previously^23^,^24^, our results are in agreement with those of researchers, suggesting a shorter catheterization time and hospitalization days in the ELRP group. There was a similar overall rate of complications in the 2 groups^11^,^12^,^13^,^14^,^15^,^16^,^17^,^18^,^19^,^20^. Total complication rates generally include bleeding, intra-abdominal organ and vessel injuries, urinary leakage, ileus, anastomotic leakage, pulmonitis, lymphocele formation, and stricture at vesicourethral anastomosis. Certainly, we also thought that the rate of complications may be associated with surgical experience and skill. Complication rates were reported by research centers, and revealed a mean complication rate of approximately 10% for the each approach^25^,^26^. Results of the current study showed a total complication rate of 11.4% (86/753) in the TLRP group, and 9.8% (61/623) in the ELRP group; the difference was not statistically significant, consistent with the findings of the above wor^25^,^26^.

Overall, it is generally considered that the main advantages of the TLRP procedure are; (1) the faster placement of trocars; and (2) the larger cavity, which allows placement of the specimen bag out of the operative field, improving vision and facilitating the vesicourethral anastomosis. However, TLRP also has many disadvantages, including the requirement of a much steeper Trendelenburg position to move the bowel out of the pelvic cavity. This position might cause upper airway and facial swelling, which may postpone extubation, lengthen the recovery time, and increase the risk of brachial plexus injury^27^. The bowels may adhere in the retropubic space of Retzius after the operation, and radiation therapy can lead to radiation enteritis. Such factors might explain the longer catheterization time and longer duration of hospitalization days associated with TLRP.

The ELRP approach has several advantages; the procedure can be finished extraperitoneally and the Trendelenburg required is less steep, compared with the transperitoneal route. The incidence rate of bowel lesions, ileus, and peritonitis is therefore lower, and the procedure prevents herniation from the trocar ports. The peritoneum can isolate the operative field from the abdominal cavity, bleeding does not contact the bowel and reflex ileus is avoided; even a poor anastomosis could not result in urinary ascites with its associated complications^8^,^9^. In addition, a self-made gasbag is inserted sufflating gas to build a pneumopreperitoneum. This keeps pressure on the peripheral tissues, and inhibits serious bleeding. However, there is also a greater risk of rectal injury with the initial dissection of the seminal vesicles, especially in obese patients. Therefore, from the above-mentioned technical considerations, ELRP might have lower blood transfusion rate and shorter intestinal function recovery time, but a similar total complication rate.

Our meta-analysis detected heterogeneity in the operation time, intraoperative blood loss, postoperative catheterization time, postoperative intestinal function recovery time, and hospitalization days. We maintain that these heterogeneities might have resulted from differences in the skill of the surgeon. Additionally, the small sample sizes, and difference between patients, which included different clinical stages and prostate volume, could have potentially increased the degree of heterogeneity. Additional factors that have been predicted to potentially amplify heterogeneity between studies include differences in country, follow-up periods, and a lack of uniformity in surgical procedure measurement standards.

Our systematic review and meta-analysis has several limitations. The study quality estimation was influenced by the non-randomized studies, and there was inadequate information provided in terms of methodological differences among the included studies. Some studies did not adequately report the outcomes measures. In addition, we could not obtain some relevant data, which may have introduced bias. Nonetheless, with the exception of operation time, the all conclusions were stable and were not impacted by the sensitivity analysis, in which each study was sequentially excluded from the pooled analysis.

In summary, both ELRP and TLRP have advantages and disadvantages; the efficacy and safety of LRP related to standardization of the procedure and the personal experience of the surgeon. However, this present meta-analysis has demonstrated that ELRP is associated with a lower blood transfusion rate, shorter catheterization time, faster intestinal function recovery time, and shorter hospitalization days, compared with TLRP. In addition, the approaches are similar in terms of operation time, intraoperative blood loss, and total complication rate. Therefore, the present meta-analysis showed that ELRP is likely to be a safe and feasible alternative for patients with localized PCa.

## Methods

### Search strategy

We performed a systematic literature search up to May 1, 2014 using Medline, Embase, the Cochrane library, and Google Scholar databases. We did not restrict our search to articles published in English. The following search terms were used: transperitoneal; extraperitoneal; LRP; PCa; or prostatic neoplasms. We also searched the relevant references of all included studies, and manually searched urology, andrology, and oncology diseases journals for further relevant articles. The search strategy was independently performed by 2 reviewers.

### Study selection

All randomized or non-randomized controlled trials that compared TLRP with ELRP and included data on at least 1 of the pre-defined outcome measures were eligible for inclusion. Studies were excluded if they met any of the following criteria; (1) not comparative studies for TLRP versus ELRP; (2) robot-assisted radical prostatectomy; (3) study sample did not comprise PCa patients. When multiple publications from the same study or institution were available, we used the publication with the largest number of cases. Review of all titles and abstracts of the included studies was independently performed by 2 authors, and full texts were screened when necessary. Any disagreements were resolved in consultation with Wei Q. All of the authors have reached a consensus with respect to included/excluded studies.

### Data extraction

The following information was recorded independently by 2 reviewers, using data extraction forms: the first author’s name, year of publication, origin country, total number of patients, average age, prostatic specific antigen level, Gleason score and the type of research design. All extracted data was cross-checked, and discrepancies in the data were resolved after discussions among all authors. The following outcome measures were recorded from the included studies: (1) operation time; (2) intraoperative blood loss; (3) blood transfusion rate; (4) postoperative catheterization time; (5) intestinal function recovery time; (6) hospitalization days; (7) total complication rate. All of the authors have reached a consensus with respect to the extracted data.

### Quality assessment

The quality of the included studies was measured independently by two reviewers using a modification of the Newcastle–Ottawa Scale^28^. Review scores ranged from 0 to 9 points for each trial. Scores ranging from 0 to 4 were defined as low-quality while those ranging from 5 to 9 were defined as high-quality. Any disagreements were resolved after discussion between the two reviewers. All of the authors have reached a consensus with respect to the quality of the included studies.

### Statistical analysis

All statistical analyses were conducted using Review Manager, version 5.1.0 (Cochrane Collaboration, Oxford, UK). Statistical analysis of dichotomous variables (blood transfusion rate and total complication rate) was performed using the OR as the summary analysis, while continuous variables (operation time, intraoperative blood loss, postoperative catheterization time, intestinal function recovery time and hospitalization days) were analyzed using the MD; accompanying 95% CI and P-value were reported. For all statistical results, P < 0.05 was considered statistically significant. The Mantel-Haenszel chi-squared test for heterogeneity was conducted. Heterogeneity was assessed using the I^2^ statistic. I^2^ values of < 50% were defined as acceptable; a fixed-effects models was used, otherwise random-effects model was applied for the meta-analysis.

## Additional Information

**How to cite this article**: De Hong, C. *et al.* Comparison of efficacy and safety of conventional laparoscopic radical prostatectomy by the transperitoneal versus extraperitoneal procedure. *Sci. Rep.* **5**, 14442; doi: 10.1038/srep14442 (2015).

## Figures and Tables

**Table 1:** Basic features and quality assessments of the included studies.

Study, year	Studydesign	Origincountry	TLRP/ELRP				Qualitylevels
			No. ofPatients	Mean age (range) (years)	Mean PSA (ng/mL)	Mean Gleason score	
Eden,^2004^	CCT	England	100/100	62.3(52–72)/61.4(45–73)	7.7(2.0–27.0)/7.6(1.0–19.0)	5.9(4.0–8.0)/6.1(4.0–9.0)	high
Cathelineau,^2004^	CCT	France	100/100	63(49–76)/61 (48–75)	8.9 ± 4.7/10.0 ± 8.0	NA	high
Ruiz,^2004^	CCT	France	165/165	64.1 ± 6.4/62.9 ± 6.8	10.8 ± 9.2/9.9 ± 8.7	5.7 ± 1.2/6.2 ± 1.0	high
Erdogru,^2004^	CCT	Germany	53/53	62.9 ± 5.4/62.9 ± 5.5	7.6 ± 3.8/7.4 ± 4.6	6.1 ± 0.8/6.0 ± 0.7	high
Remzi,^2005^	CCT	Austria	39/41	61.0 ± 11.0/59.0 ± 12.0	5.5 ± 3.7/8.1 ± 6.1	5.1 ± 1.2/5.5 ± 1.3	high
Brown,^2005^	CCT	America	122/34	58/56	NA	NA	high
Porpiglia,^2006^	CCT	Italy	80/80	64.2 ± 5.1/64.4 ± 5.9	8.3 ± 4.3/9.7 ± 5.7	NA	high
Katz,^2006^	CCT	France	22/13	67.5 ± 4.4/67.5 ± 4.4	NA	NA	high
Gao,^2006^	CCT	China	12/19	62.6 ± 6.7/63.1 ± 7.7	9.7 ± 3.1/8.8 ± 1.8	NA	high
Wang,^2008^	CCT	China	21/12	66(46–74)/66(56–73)	28.9(9–120)/32.2(5–130)	6.6(5.0–9.0)/7.0(6.0–9.0)	high
Siqueira,^2010^	CCT	Brazil	40/40	59.8 ± 6.8/63.6 ± 7.9	5.4 ± 2.0/5.9 ± 1.9	NA	high
Wang,^2010^	CCT	China	39/15	68.1 ± 5.2/68.3 ± 6.1	15.4 ± 4.2/14.1 ± 6.3	7.4 ± 0.8/7.2 ± 0.7	high
San,^2012^	CCT	China	30/30	65.8(45–73)/66.1(42–70)	36.2(5–130)/ 36.2(5–130)	7.0(6.0–9.0)/7.0(6.0–9.0)	high
Zhao,^2014^	CCT	China	116/74	66.8(50–78)/ 66.8(50–78)	11.13(1.13–28.35)/11.13(1.13–28.35)	NA	high

TLRP = transperitoneal laparoscopic radical prostatectomy; ELRP=extraperitoneal laparoscopic radical prostatectomy; PSA = prostate specific antigen; NA = not applicable; CCT = clinical controlled trial.
